# A PROGRESS-driven approach to cognitive outcomes after traumatic brain injury: A study protocol for advancing equity, diversity, and inclusion through knowledge synthesis and mobilization

**DOI:** 10.1371/journal.pone.0307418

**Published:** 2024-07-22

**Authors:** Thaisa Tylinski Sant’Ana, Sara Hanafy, Esme Fuller-Thomson, Michelle McDonald, Angela Colantonio, Daìthì Cee, Gráinne McGettrick, Brian Lawlor, Tatyana Mollayeva

**Affiliations:** 1 KITE-Toronto Rehabilitation Institute, University Health Network, Toronto, Ontario, Canada; 2 Acquired Brain Injury Research Lab, University of Toronto, Toronto, Ontario, Canada; 3 Rehabilitation Sciences Institute, Temerty Faculty of Medicine, University of Toronto, Toronto, Ontario, Canada; 4 Institute for Life Course & Aging, University of Toronto, Toronto, Ontario, Canada; 5 Factor-Inwentash Faculty of Social Work, University of Toronto, Toronto, Ontario, Canada; 6 Department of Family & Community Medicine, Temerty Faculty of Medicine, University of Toronto, Toronto, Ontario, Canada; 7 Faculty of Nursing, University of Toronto, Toronto, Ontario, Canada; 8 Brain Injury Canada, Ottawa, Ontario, Canada; 9 Department of Occupational Science & Occupational Therapy, Temerty Faculty of Medicine, University of Toronto, Toronto, Ontario, Canada; 10 Dalla Lana School of Public Health, University of Toronto, University of Toronto, Toronto, Ontario, Canada; 11 Flemish Dementia Working Group, Publications Department, Aalter, East Flanders, Belgium; 12 Global Brain Health Institute, University of California San Francisco, San Francisco, Francisco, California, United States of America; 13 Acquired Brain Injury Ireland, Dublin, Leinster, Ireland; 14 Trinity College Dublin, University of Dublin, Dublin, Leinster, Ireland; Public Library of Science, UNITED STATES OF AMERICA

## Abstract

Evidence syntheses for advancing equitable traumatic brain injury (TBI) research, policy, and practice presents formidable challenges. Research and clinical frameworks are currently not specific to equity, diversity, and inclusion considerations, despite evidence that persons with TBI live in societies in which power imbalances and systems of social dominance may privilege some people and marginalize others. The present protocol outlines a strategy for a research program, supported by the Canadian Institutes of Health Research, that explores the integration of PROGRESS-Plus parameters in research with the goal of advancing open-science databases and tools to improve our understanding of equity in cognitive and brain health outcomes in TBI. PROGRESS-Plus is a framework outlining social, economic, and cultural parameters that may influence health opportunities and outcomes (e.g., place of residence, race, occupation, gender, etc.). A multistep research program is proposed to support three objectives: (1) organizing existing data on TBI-induced changes in cognition and brain health into a template to facilitate future research, including research using machine learning techniques; (2) updating published evidence with a more rigorous approach to the consideration of PROGRESS-Plus parameters; and (3) mobilizing knowledge on the current state of evidence that is relevant, equitable, and accessible. This program facilitates partnerships with knowledge users across clinical, research, academic, and community sectors to address the three research objectives through a unifying workflow of exchange, synthesis, and knowledge mobilization. We anticipate that this global collaboration between topic experts and community leaders in equity in brain health will add significant value to the field of TBI by promoting equity-transformative advancements in knowledge synthesis, policy, and practice.

## Introduction

Consideration of social equity parameters in evidence synthesis is essential to provide relevant, timely, and quality data to inform equitable policy design, implementation, evaluation and decision making [1–3]. Active integration of such parameters in health research [4] can begin to untangle the unfair practices that perpetuate inequities in brain health outcomes, driven by systems of social dominance that privilege some groups while marginalizing others [5, 6]. Although definitions of brain health vary, most refer to a lifelong dynamic state of cognitive, emotional, and motor capacities, underpinned by physiological processes that change with age [7]. One of the most significant threats to brain health is traumatic brain injury (TBI), an injury to the brain caused by an external force to the head or body [8]. The annual global incidence of TBI is enormous; it is more common than breast cancer, HIV/AIDS, spinal cord injury, and multiple sclerosis combined [9, 10]. Traumatic brain injury is regarded as one of the most costly neurological conditions in Canada, with estimated indirect economic costs amounting to $8.2 billion by 2031 [11], not taking into account the emotional burden it places on men, women, and gender-diverse people who struggle to fulfill familial and social roles after brain injury.

Our recent evidence syntheses of international studies (systematic review and meta-analyses) concerning the course and prognostic factors of cognitive and other brain-health related outcomes in TBI highlighted non-uniform prognoses for people with the same injury severity, even after controlling for a number of known risk factors affecting brain health [12, 13]. Our recent population-based research conducted with data from Ontario, the largest Canadian province, highlighted that pre-injury brain health, access to healthcare, health-seeking behaviours, time dedicated to health-promoting practices, and self-care is not equal between and within different social groups, despite Canada’s publicly funded healthcare system [14–17]. Furthermore, the findings of these same studies also suggested that brain injury often exacerbates such health and social inequities.

When it comes to raising awareness and increasing understanding around social equity parameters that drive differences in brain health outcomes, TBI is still within relatively unchartered territory, emphasizing the need to adapt scientific evidence on the effects of social parameters to inform equity-driven policy and practices. In recognition of this need, a novel operating grant titled “A PROGRESS-Driven Approach to Cognitive Outcomes after Traumatic Brain Injury: Advancing Equity, Diversity, and Inclusion through Knowledge Synthesis and Mobilization” was proposed by our international team with expertise in knowledge translation, sex- and gender-based analysis, equity in brain health, intersectionality, brain injury medicine, rehabilitation science, psychology, social theory, safety science and behavioral change. The team adhered to critical feedback from reviewers of Canadian Institutes of Health Research (CIHR) sex- and gender-sensitive programs [18] and internal ethics review groups to ensure alignment of the research proposal with the CIHR’s position on Equity, Diversity, and Inclusion (EDI) [19] and the Canadian Government’s call for Sex- and Gender-Based Analysis Plus (SGBA+), an analytical approach to examine the intersecting effects of sex, gender, and other factors (e.g. age, race, etc.) to promote equity [20]. Our research will investigate equity in brain health outcomes using PROGRESS-Plus, a framework that identifies social, economic, and cultural parameters that may influence health opportunities and outcomes (e.g., place of residence, race, occupation, gender, etc.) [21].

**Fig 1:**
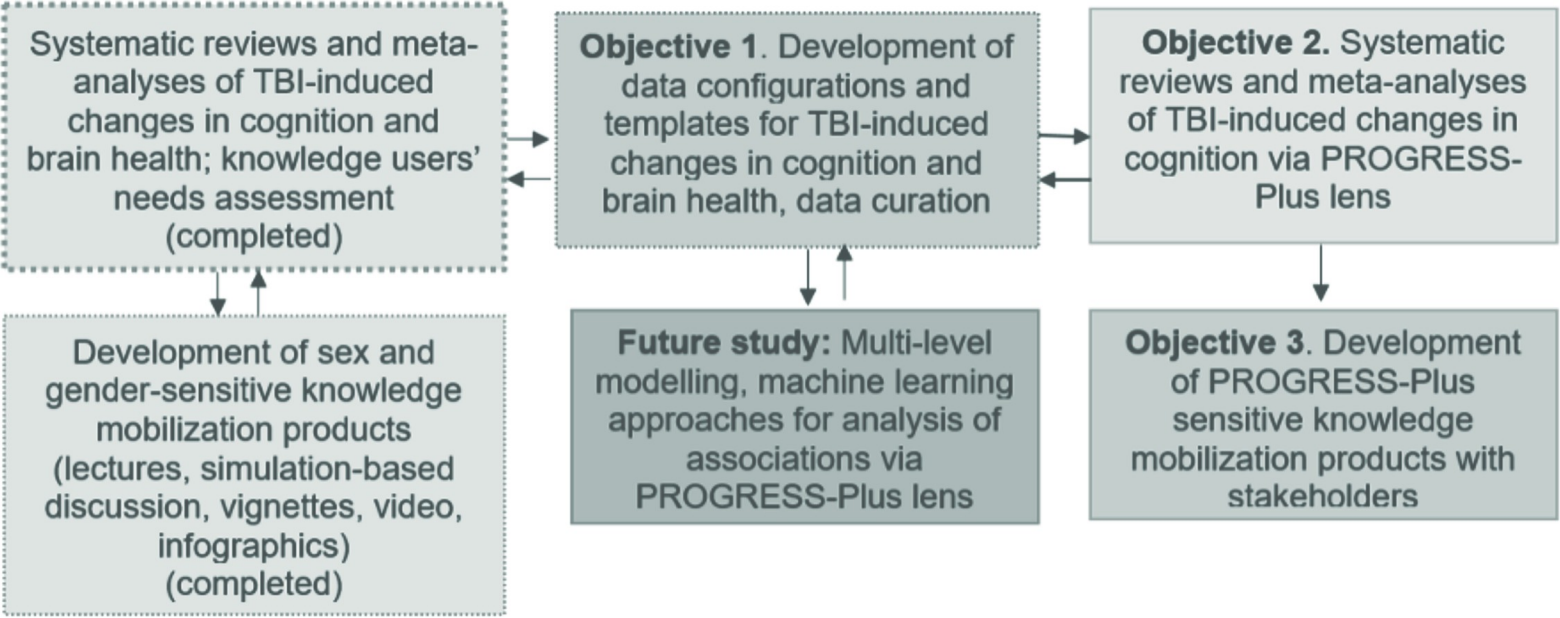
Research workflow and relationship between the objectives.

Through the publication of this protocol, we aim to strengthen the scientific rigor of the proposed research program by creating an early scientific record of the study’s methodology. The overarching goal of this research is to advance Canadian research excellence on brain health equity for people with TBI, and to ensure maximum impact through global collaboration with diverse stakeholders and knowledge users. To achieve this goal, our research objectives are three-fold (Fig 1):

1. To organize existing data on TBI-induced changes in cognition and brain health for research, specifically for projects using machine learning techniques, and to develop a template to facilitate periodic update of the databank;
2. To update our prior knowledge syntheses [12, 13] with a more rigorous approach to social equity parameters, utilizing the PROGRESS-Plus framework [21] and recommendations of the Evidence for Policy & Practice Information Centre [22]; and
3. To conduct knowledge translation and mobilization efforts that are relevant to substantive equality in brain health outcomes. Substantive equality refers to the achievement of equal outcomes through equal access to services and benefits that meet each persons’ unique needs [23].

## Materials and methods

### Ethics

We obtained research ethics approval from the ethics committees at the clinical institution at which the research is taking place (University Health Network, UHN REB 24–5397). For the survey part of the Objective 2’s priority setting process, research participants will provide an informed consent (S4 File), confirming that they: (1) understood the essential elements of the research, including risks and benefits, and (2) consent to participate. The ethics board waived the requirement for a signed consent form. For more details on the informed consent process, please refer to the priority setting process section of the protocol.

### Protocol registration

We will register this study on Open Science Framework (OSF) [24], a research project management tool to promote transparency in the research process. Our previously published systematic reviews [12, 13] are registered on PROSPERO (CRD42017055309, CRD42018098697). We created new registries for the updated reviews to reflect the substantial shift of focus to incorporate PROGRESS-Plus parameters in the present study. Protocols were registered on PROSPERO on May 17, 2024 (CRD42024547415, CRD42024547456). Reporting of this protocol followed PRISMA-P guidelines (S1 File).

### Study conduct and reporting

We will follow the FAIR Guiding Principles [25], which state that research should be Findable, Accessible, Interoperable, and Reusable, to support replicability and transparency of the research process and maximize opportunity for further applications of data and data templates. To reduce costs and streamline our research process, we are exploring implementation of crowdsourcing [26], natural language processing and machine learning [27], and research/project management tools, such as the systematic review management tool Covidence [28].

### Patient and public involvement

This protocol was informed by input from patient and public representatives. We will follow best practices for patient and public involvement in research [29], enabling equitable and shared decision-making with people with lived experience and the public. We collaborated with partner organizations and people with lived experience during grant development and will continue to do so throughout the entire research process. Applying a knowledge-user feedback framework [29], current scientific evidence, and the team’s expertise, all concepts and ideas emerging in the research process will be documented and incorporated.

People with lived experience who are part of our research team (Table 1) will participate in bi-monthly team meetings, contributing to the research process and end-of-grant knowledge translation. Individuals with lived experience will present their unique perspectives and preferences, which we expect will facilitate operationalization of person-specific PROGRESS-Plus components and tailoring of knowledge translation materials for knowledge users. For the purposes of this research, a knowledge user is defined as “an individual who is likely to be able to use research results to make informed decisions about health policies, programs and/or practices” [30].

**Table 1:** Team members’ affiliations, expertise, and roles within the research project.

Location	Affiliations/organization/network: Expertise	Team Member, Role(s)
Toronto, Canada	University of Toronto/University Health Network/Global Brain Health Institute/Canadian Consortium on Neurodegeneration in Aging: acquired brain injury; equity in brain health; social determinants of health; public health; knowledge translation; Alzheimer’s disease; dementia prevention; health & social policy; neurology; acquired brain injury; preventive medicine; cognitive rehabilitation; research methods; evidence synthesis; disability; gender-based analysis; health status transitions	Tatyana Mollayeva, NPA
	University of Toronto/University Health Network/Acquired Brain Injury Lab University of Toronto: women; sex and gender; work-related traumatic brain injury; older adults; under-served populations; rehabilitation; vulnerable populations; research-informed theatre; knowledge translation; epidemiology	Angela Colantonio, Co-A
	University of Toronto/Canadian Consortium on Neurodegeneration in Aging: women’s brain health and aging; dementia; culture and experience; women’s health; cognitive neuroscience; integrated knowledge translation; sex differences; gender and health; interactions between sex and gender psychology; knowledge mobilization	Gillian Einstein, Co-A, Partner, Knowledge User
	University of Toronto/University Health Network: knowledge translation; rehabilitation sciences; mixed methods; transitions in care; knowledge synthesis; self-management; peer support; vocational rehabilitation; acquired brain injury; policy and evaluation	Sara Hanafy, Co-A
	University of Toronto: biostatistics; computer science; artificial intelligence; machine learning; sex and gender-based analysis; theoretical and applied statistical analyses; modeling population heterogeneity; applied Bayesian method	Michael Escobar, Co-A
	University of Toronto/ Institute for Life Course and Aging: social determinants of health; gerontology; chronic health conditions; dementia;mental health; adverse childhood experiences; resilience; life course and aging; maltreatment; evidence-based practice; research methods; knowledge translation	Esme Fuller-Thomson, Co-A, Partner, Knowledge User
	University of Toronto: rehabilitation; neurorehabilitation; knowledge translation; ageing; capacity building; disability; health information product development; technology; community building; advocacy	Milos Popovic, Partner, Knowledge User
	Brain Injury Canada: knowledge facilitation; knowledge translation; strategic planning; injury management; acquired brain injury; advocacy; health information product development; arts in sociology; neurorehabilitation	Michelle McDonald, Co-A, Partner, Knowledge User
	KITE-Toronto Rehabilitation Institute: science communication; knowledge translation; health information product development	Thaisa Tylinski Sant’Ana, Research Analyst
Dublin, Ireland	Global Brain Health Institute/ Trinity College Dublin: Alzheimer’s disease; biomarkers; carers & caregiving; outreach & education; dementia care; dementia prevention; psychiatry; mental health; brain health; old age; global equity in brain health	Brian Lawlor, Co-A, Partner, Knowledge User
	Acquired Brain Injury Ireland/ Global Brain Health Institute/Trinity College Dublin: acquired brain injury; social determinants of health; advocacy; gender-diversity; research and policy management; carers & caregiving; brain injury care; health & social policy; equity in brain health; social sciences	Gráinne McGettrick, Co-A, Partner, Knowledge User
Leeds/ London, UK	Global Brain Health Institute/ Freelance Artist: equity in brain health, cognition; dementia prevention; traumatic brain injury; lived experience; theatre; creative art; narrative story; healthy aging; health disparities; creator of spaces that promote dialogue	Dominic Gately, Co-A, Person with lived experience
Aalter, Belgium	Flemish Dementia Working Group in Belgium/LGBTQ+ Dementia UK: advocacy; LGBTQ+ Dementia Advisor; education; gender-diversity; personal experience of dementia; dementia care; health & social policy; gender inequalities; equity in brain health	Mx. Daìthì Clayton (Cee), Co-A, Person with lived experience

Abbreviations: Co-A, co-applicant; NP, nominated principal applicant

### Objective 1: Organizing data and developing templates for ongoing data collection

To incorporate a PROGRESS-Plus lens to the knowledge synthesis on TBI-induced cognitive changes and brain health outcomes, we will develop 1) metadata configurations; 2) templates to standardize data collection; and 3) protocols for data curation and upload. Our two previous systematic reviews on cognitive changes [12] and health- and function-related outcomes of persons with TBI of various injury severities [13] featured 36 and 58 studies, respectively. We will perform a full-text review of these studies to determine to what extent PROGRESS-Plus variables were considered by the authors. We will aggregate data with emphasis on PROGRESS-specific variables and organize the data in a repository that will serve as a template to facilitate further analysis (e.g., machine learning analyses, including classical and Bayesian meta-analysis, multi-level modeling, and AI-driven approaches).

### Objective 2: Updated knowledge synthesis

The process of study selection, data management, extraction and synthesis, risk of bias assessment, and reporting will follow the methodology of our previous systematic reviews [12, 13] outlined in detail in the published protocols [31, 32]. Briefly, for the systematic review of cognitive changes [12], we included peer-reviewed, English language studies that assessed cognition by clinical diagnosis or standardized measure, targeted adult patients with a TBI determined by clinical evaluation, and followed them for any period of time. We excluded treated cohorts from studies of intervention efficacy for cognitive performance, utilizing data from untreated cohorts only. For the systematic review on health- and function-related outcomes [13], we included original peer-reviewed English language studies of all designs that (1) included adults with TBI diagnosis made by clinicians or specialists; (2) adequately described at least three key characteristics of their participants: age, sex, and severity of TBI; and (3) provided sex/gender-stratified results, taking into account at least age and TBI severity; or (4) included sex/gender or an interaction between sex and other variable(s) as a covariate in their statistical analysis of any TBI outcome, along with at least age and TBI severity; or (5) qualitatively discussed implications of sex/gender on TBI outcomes, considering age and injury severity. For this systematic review, we excluded studies that (1) did not specify how a TBI diagnosis was made; (2) focused on TBI itself as an outcome; (3) compared TBI samples with other clinical populations without evaluating the effect of sex/gender in TBI separately; or (4) focused on sports-related concussion. We excluded studies on sports-related concussion to avoid redundancy due to other published systematic reviews on the topic [33–35]. Case reports, paediatric studies, dissertations, articles with no primary data, single-sex studies, studies that did not consider TBI severity and age were excluded from both systematic reviews.

In our previous review on cognitive changes, we focused on five domains of cognitive functioning: recent memory, language, visuo-spatial ability, executive function, and information processing speed. For the update, we will include an additional category of social cognition [36]. For the review on health- and function-related outcomes, we considered the following categories: mortality, structural/physiological, care, medical, psychiatric disorders, sleep-related, cognitive functioning, functional outcome, disability, social participation, work-related, and life satisfaction. For the update on the reviews, we will exclude categories that do not fall within the construct of brain health, as defined by global organizations [7] and subsequently rated by research team members. The working definition of brain health prioritized by the team is “state of brain function across cognitive, sensory, socio-emotional, behavioural, and motor domains” [37] (S1 Table and S1 Fig). Thus, outcomes concerning mortality and care will be excluded. The scope of the systematic reviews is summarized in Table 2.

**Table 2:** Frameworks used to determine scope of systematic reviews. For quantitative studies, we will use the Participants, Interventions, Comparators, Outcomes, Study type (PICOS) framework. For qualitative studies, we will use Participants, Issue, and Evaluation/Effect (PIE) framework.

PICOS Framework	
Participants	Adults with traumatic brain injury
Interventions	Not applicable
Comparators	Any PROGRESS-Plus parameters reported in analysis by study authors
Outcomes	Brain health outcomes (i.e. structural/physiological, medical, psychiatric disorders, sleep-related, cognitive functioning, functional outcome, disability, social participation, work-related, and life satisfaction)
Study type	Observational studies
**PIE Framework**	
Participants	Adults with traumatic brain injury
Issue	Extent of consideration of PROGRESS-Plus parameters
Evaluation/Effect	Brain health-related experiences, perceptions, and behaviors

We repeated searches on the two systematic reviews in March and April 2024 (S2 and S3 Files). Two independent researchers will screen for eligibility and inclusion, and any disagreements will be resolved through discussions with the senior author. For the update on the systematic reviews, the following data will be extracted using templates developed in previous reviews [12, 13]: (1) study information (i.e., authors, publication year, country, Gender Inequality Index (GII) of country of study origin, objectives, design, sample size, outcomes assessed and measures used, and whether effect of PROGRESS-Plus variables was investigated); (2) participant TBI-related variables (i.e., injury severity, external cause of injury, time since injury/stage post injury, outcomes concerning brain health, etc.); and (3) participant social equity information (i.e., PROGRESS-Plus variables) (Table 3). In case of unclear data, authors of the original study will be contacted.

**Table 3:** Preliminary categories to be included in the PROGRESS-Plus data collection [38, 39].

PROGRESS	
Place of residence	Housing characteristics; insecurity/instability; neighbourhood; rurality; geographic area; etc.
Race, ethnicity, culture, and language	Race, ethnic, and/or cultural background; shared language; etc.
Occupation	Employment status and type; industry; unionization; job security; workplace health and safety; etc.
Gender and/or biological sex	Sex at birth; gender of person [40]
Religion	Religious background, lack of religion
Education	Level of education; major field of study; type of institution; informal education; etc.
Social capital	Marital status; trust in relationship; social network size; community participation; etc.
Socioeconomic position (SEP)	Income level; wealth and assets; etc.
**Plus**	
All SEP	SEP income related, plus occupation, education, and place of residence
Age	Chronological and biological (including stage of development)
(Dis)ability	Physical; mental; developmental; emotional status; disability; etc.
Sexual Orientation	Sexual orientation (e.g. gay, lesbian, heterosexual, etc.)
Other Parameters	Imprisonment; exposure to assault/abuse; etc.

The data extraction process will consist of three phases. In the first phase, we will extract all PROGRESS-Plus variables from included studies, along with data type and profile (structure, format, unique values, etc.). In the second phase, we will create a separate matrix for each PROGRESS-Plus variable. The columns will represent each extracted variable and its measure, while the rows will represent the included publications that considered this variable. We will use crosses to indicate similar measures used and color to mark studies concerning similar brain injury and brain health outcomes. In the final phase, we will identify the frequency order of the considered PROGRESS-Plus variables for each injury severity and brain health related outcomes separately. For this, we will replace the crosses of the matrix with ascending numbers.

Next, we will discuss data transformation and harmonization. We expect the data harmonization process to follow the steps proposed by Kumar et al [41], but is subject to change based on the results of data extraction. We will summarize data extraction results, by PROGRESS-Plus parameter, for cognition and brain health outcomes by injury severity and phase of assessment after the injury, if data permit. We will organize results according to the approach taken to investigate the association between PROGRESS-Plus variables and studied outcomes. In situations where sufficient homogeneity is achieved, we will pool the data for similar outcomes stratified by subgroups of sex/gender, injury severity, and phase of assessment after the injury in meta-regression following methodologies suggested by Cochrane [42]. If pooling is not possible, we will use analytical approaches to data analysis as suggested by Slavin [43].

#### Priority setting process

As part of Objective 2, we will conduct a priority setting process engaging five stakeholder groups: (1) persons with lived experience, (2) family/friends, (3) healthcare providers, (4) researchers, and (5) staff/leaders in brain health organizations. Our target is to invite participants who represent a diverse range of gender identities, races/ethnicities, specializations, career stages, professional roles, and affiliations. We estimate the need to survey at least 10 persons per stakeholder group, amounting to at least 50 persons (i.e., 10x5). Stakeholders can belong to more than one stakeholder group and, in that case, they will be counted as belonging to the least represented group. The proposed sample size is consistent with that deemed usual for sampling in a relatively homogeneous group of stakeholders in term of the injury (i.e., TBI), and should provide a sufficient number of participants to cover the points of interest for stakeholders in a priority setting activity [44–46].

To meet criteria of the informed consent, we created a participant information sheet that addresses the purpose and objectives of the research, participant eligibility, risks and benefits of participating, and information about withdrawing from the survey (S4 File). We implemented the “Question Skip Logic” feature to automatically remove (disqualify) participants who do not consent to participating in the study.

Participants will be screened for eligibility according to the following inclusion criteria: being 18 years old or older, having the ability to communicate in English, and identifying as one or more of the following: person with traumatic brain injury; family member or friend who assists a person with traumatic brain injury with any activity of daily living (i.e., meal preparation, home maintenance, etc.) or provides emotional support without financial compensation; researcher directly involved in traumatic brain injury research; health care professional who provides care for people with traumatic brain injury and works with them at least part-time; person holding a staff or leadership position in an organization directly representing or serving people with traumatic brain injury and their families.

Participants will be invited to complete an online survey on Survey Monkey. It contains questions about participants’ age range, sex at birth, gender, race, occupation, country of residence, and whether you live in a rural or urban/suburban area. Participants will then be presented with a list of PROGRESS-Plus factors linked to cognitive and brain health outcomes, and will be asked to rank list items by perceived level of importance to equity progress using a 4-point scale (1—not important; 4—very important). The survey also asks participants to reflect on positive and negative impacts of PROGRESS-Plus parameters on their personal/professional experiences with TBI, and to suggest strategies to incorporate these parameters into research, policy, and/or practice. Finally, the survey asks participants to elaborate on helpful information/resources for people with lived experience of TBI and their support system, and the format of delivery for such information. We will use ranking results and qualitative responses to inform knowledge mobilization initiatives.

### Objective 3: Knowledge mobilization that is relevant to the substantive equality

**Fig 2:**
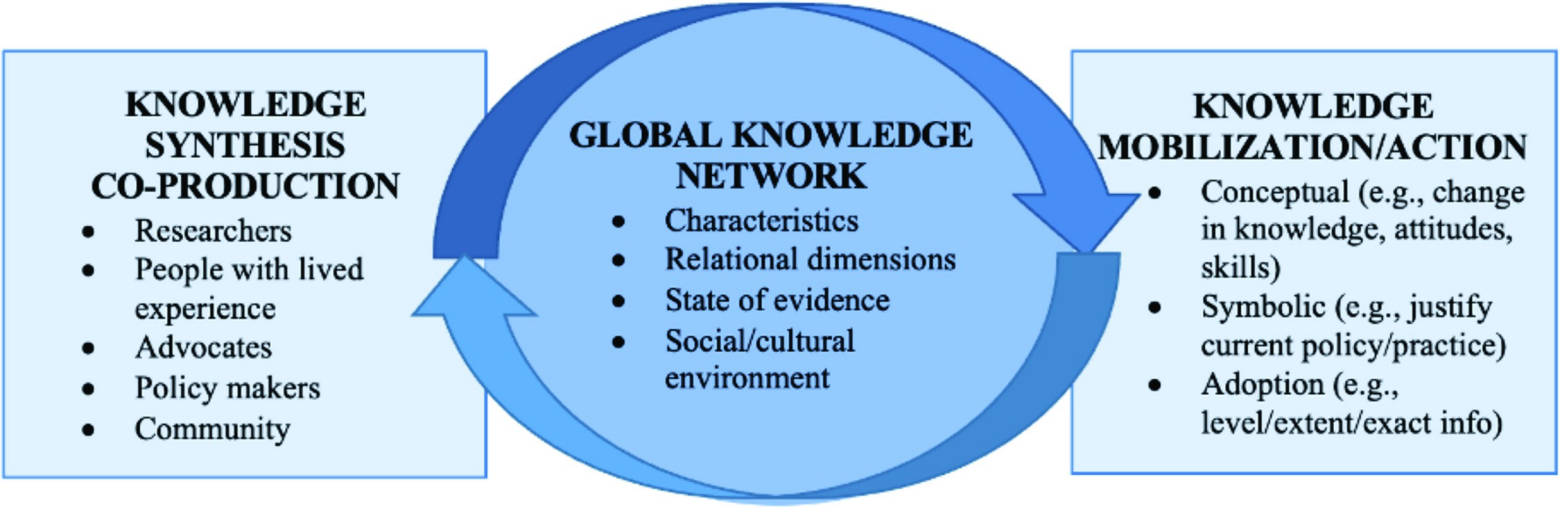
The knowledge-action framework for the proposed research. Adopted from CIHR’s Knowledge Mobilization Framework and Action Plan. The framework captures the dynamic processes of knowledge synthesis and mobilization in the development of our proposed products.

A Round Robin activity [47, 48] will be conducted with internal stakeholders (i.e., team members) to discuss knowledge mobilization and translation initiatives that reflect users’ priorities, preferences, and needs expressed in the online survey. This is essential since knowledge communicated in peer-reviewed journals might not reach individuals living with TBI, their family members and friends, brain health advocates, clinicians, and policymakers. We will continue to work alongside relevant stakeholders including individuals with TBI for this objective, integrating their insights on the knowledge synthesis results to develop the content for our knowledge mobilization products (Fig 2).

#### Round robin activity procedure

A summary of the results of the priority setting survey (Objective 2) will be shared with the research team. We will ask each team member to prepare two ideas of knowledge translation tools and/or mobilization strategies that focus on the top PROGRESS-Plus parameter(s) identified by external stakeholders. We will ask them to submit their ideas to the Study Coordinator and the PI a few days prior to the meeting to compile them into a single file which will be shared with team members the day before the meeting. The procedure is described elsewhere [49].

Team members will meet on an online video conferencing platform (e.g., Zoom) to participate in the Round Robin activity [47, 48]. Each participant will be asked to describe their idea in more detail and, as this happens, other members will be encouraged to build upon and provide feedback. When all team members have shared their ideas, we will initiate a discussion around feasibility, potential impact, and implementation strategies, and we will identify recurring themes, promising concepts, and areas for further development. At the end of the discussion, each team member will fill out a poll to rank the ideas shared during the activity to identify the top knowledge translation tool(s) and mobilization strategies that will be developed as part of the research project. We estimate the Round Robin activity to last for three hours.

#### Knowledge dissemination

Finally, we will utilize a variety of methods to disseminate the outputs of the research, including: (i) sharing a final report with the leadership of the UHN, Canadian Consortium on Neurodegeneration in Aging [50], Brain Injury Canada [51], Acquired Brain Injury Ireland [52], and the Global Brain Health Institute [53]; (ii) offering links to publications and emerging knowledge mobilization products to national and international brain injury and brain health organizations/agencies; (iii) publishing paper(s) summarizing the results in scientific journals; (iv) presenting at seminars, professional association meetings, and conferences, and (v) using social media to disseminate findings to the general public. Bilingual team members will translate key findings from English to other languages to reach a broad, global audience.

## Discussion

The study of social parameters in TBI research is important to promote policy and practices that advance brain health equity. However, the topic of integration of social parameters in brain health research has not been systematically reviewed to date, preventing the potentially valuable knowledge from reaching knowledge users. This protocol intends to fill this gap by systematizing research data and feedback from people with lived experience of TBI to ensure value of knowledge synthesis and mobilization to its knowledge users. We anticipate that our efforts over the course of one year will capture the current extent of PROGRESS-Plus consideration in TBI-related brain outcomes, prioritize the development of templates for future research utilizing machine learning techniques, and motivate the initiation of PROGRESS-Plus data transformation and harmonization. While each of these outputs is significant in informing equity considerations, further subgroup analyses, identification of knowledge gaps, and complete data harmonization and transformation of PROGRESS-Plus variables is essential and would require future grant applications, funding, and targeted research.

For updates on searches, we aim to follow published guidelines and recommendations for projects including living evidence collection [54–56]. We plan to repeat searches every year from now, including screening for abstracts of the newly retrieved reports, contingent upon future funding to sustain the project. The annual interval for screening was chosen because we expect no sudden rise in relevant publications that could justify more frequent screening. The evidence syntheses will be updated every third year, providing that a sufficient quantity of new records is identified for inclusion, and we obtain further funding to continue this research. As a threshold for updating evidence syntheses, we plan to use 50 new records, but we will consider updating the evidence earlier if ground-breaking PROGRESS-Plus evidence is published. We define ground-breaking PROGRESS-Plus evidence as, for example, the publication of a measure/tool or procedure that is immediately accessible to researchers conducting systematic reviews concerning equity [57], and which offers substantial automation of the data extraction process, or a measure/tool or procedure that aims to change the existing systematic review recommendations towards one or more of the following: (i) definition of equity; (ii) hypotheses related to equity and logic models; (iii) appropriate study designs; (iv) appropriate outcomes; and (v) context.

Our multidisciplinary team has close ties to communities of diverse backgrounds, offering valuable opportunities to disseminate findings to knowledge users and decision-makers across the globe. Our team members are also part of multiple consortia, professional organizations, and networks, including the North American Brain Injury Society, Canadian Traumatic Brain Injury Consortium, American Congress of Rehabilitation Medicine, and the Global Brain Health Institute, each of which has its own knowledge brokers to facilitate knowledge translation and exchange support to reach a global audience. We anticipate that this research will add significant value to the field of TBI by promoting equity-transformative scientific advancements in knowledge synthesis, policy, and practice to enhance access to knowledge for all.

## Supporting information

S1 Fig Attributes of brain health ranked by team members via online survey. A lower score represents a higher importance. Score was calculated as ((1*number of people who ranked item as #1)+(2*number of people who ranked item as #2)+…+(5*number of people who ranked item as #5))/number of respondents.
(TIFF)

S1 File PRISMA-P checklist. Preferred Reporting Items for Systematic review and Meta-Analysis Protocols (PRIMA-P) 2015: recommended items to address in a systematic review protocol.
(DOC)

S2 File Search strategies for update of systematic review on course and prognostic factors of cognitive outcomes after traumatic brain injury. (PDF)

S3 File Search strategies for update of systematic review on sex and gender effects in clinical and functional outcomes of traumatic brain injury. (PDF)

S4 File Informed consent form for participation in a research study. (PDF)

S1 Table Attributes of brain health. (PDF)
