# Correction: A Progress-driven approach to cognitive outcomes after traumatic brain injury: A study protocol for advancing equity, diversity, and inclusion through knowledge synthesis and mobilization

**DOI:** 10.1371/journal.pone.0319818

**Published:** 2025-03-05

**Authors:** The PLOS One staff

The first author initials appear incorrectly in the citation. The correct citation is: Tylinski Sant’Ana T, Hanafy S, Fuller-Thomson E, McDonald M, Colantonio A, Cee D, et al. (2024) A Progress-driven approach to cognitive outcomes after traumatic brain injury: A study protocol for advancing equity, diversity, and inclusion through knowledge synthesis and mobilization. PLoS ONE 19(7): e0307418. https://doi.org/10.1371/journal.pone.0307418.

The publisher apologizes for the error.

## References

[1] Sant’Ana TT, Hanafy S, Fuller-Thomson E, McDonald M, Colantonio A, Cee D, et al. A Progress-driven approach to cognitive outcomes after traumatic brain injury: A study protocol for advancing equity, diversity, and inclusion through knowledge synthesis and mobilization. PLoS One. 2024;19(7):e0307418. doi: 10.1371/journal.pone.0307418 39037993 PMC11262676

