# Supplementary figures and images for: A PROGRESS-driven approach to cognitive outcomes after traumatic brain injury: A study protocol for advancing equity, diversity, and inclusion through knowledge synthesis and mobilization
Source: PLoS One. 2024 Jul 22;19(7):e0307418. doi: 10.1371/journal.pone.0307418 (PMC11262676; doi:10.1371/journal.pone.0307418)

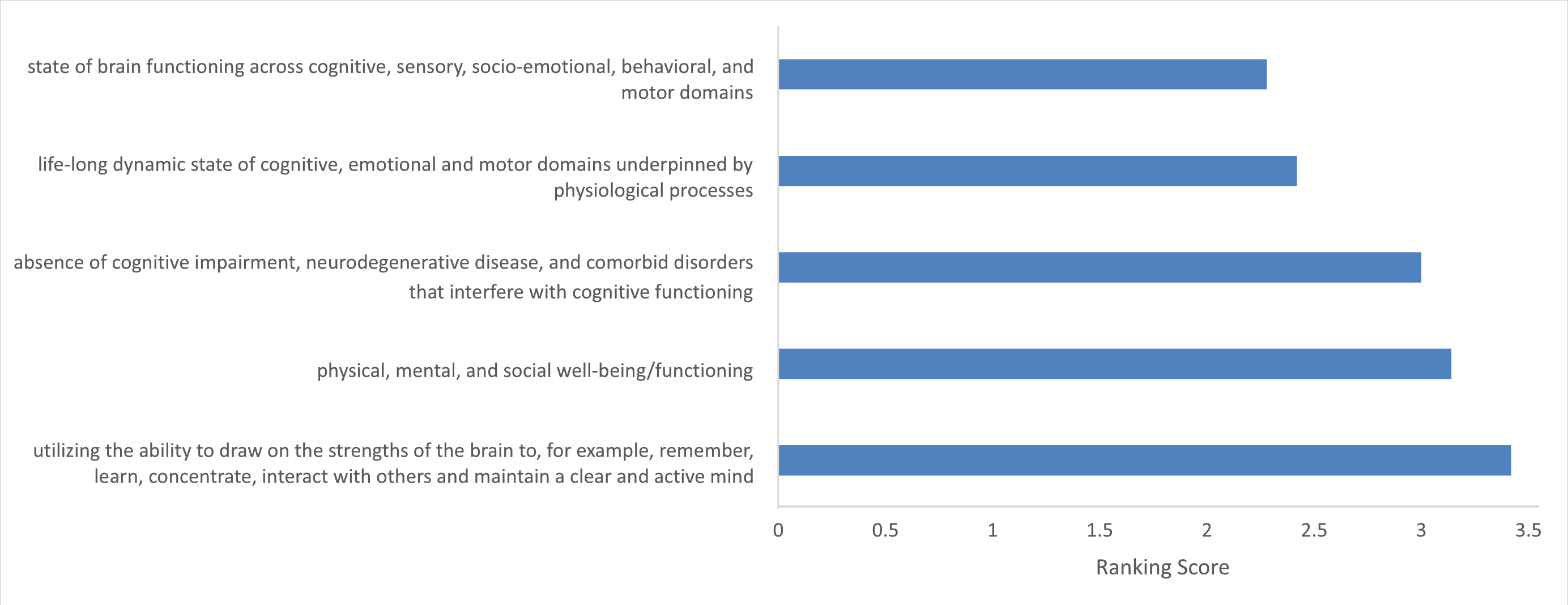

Supplement: S1 Fig — A lower score represents a higher importance. Score was calculated as ((1*number of people who ranked item as #1)+(2*number of people who ranked item as #2)+…+(5*number of people who ranked item as #5))/number of respondents. (TIFF) [file pone.0307418.s001.tiff]
