# Supplementary material for: A PROGRESS-driven approach to cognitive outcomes after traumatic brain injury: A study protocol for advancing equity, diversity, and inclusion through knowledge synthesis and mobilization
Source: PLoS One. 2024 Jul 22;19(7):e0307418. doi: 10.1371/journal.pone.0307418 (PMC11262676; doi:10.1371/journal.pone.0307418)
Supplement: S2 File — (PDF) [file pone.0307418.s003.pdf]

Supplementary File 3: Search strategies for update of systematic review on course and prognostic factors of cognitive outcomes after traumatic brain injury

SEARCH DETAILS

Completed by: Cynthia Chui, MI
Submitted: April 8, 2024

Searches were conducted in MEDLINE(R) ALL (Ovid), Embase (Ovid), Cochrane Central Register of Controlled Trials (Ovid), PsycINFO (Ovid), and SCOPUS for an update of a systematic review (doi: 10.1016/j.neubiorev.2019.01.011).

Search strategies were created by Jessica Babineau for the previous systematic review, which included the use of text words and subject headings (e.g. MeSH, Emtree) related to (1) traumatic brain injury, (2) cognitive outcomes, and (3) longitudinal studies. All searches were limited to English and were updated to include articles added to the databases from December 2016 to April 8, 2024.

The updated search strategies included minor revisions (e.g. due to updates to databases):

- The keyword search field for "kw" was changed to "kf" in Medline
- MEDLINE(R) ALL was used instead of Medline and Medline in-process, which also removed the need to do a supplemental search in PubMed (due to coverage of PubMed's citations)
- The keyword search field "kf" was added to the Embase search strategy, where appropriate and consistent with the original Medline strategy from which it was translated from

UPDATED SEARCH – NEW RESULTS PRE-DUPLICATE REMOVAL

TOTAL Results: 31,704 citations

- MEDLINE(R) ALL: 7,291 citations
- Embase: 14,205 citations
- Cochrane Central Register of Controlled Trials: 4,046 citations
- PsycINFO: 2,027 citations
- SCOPUS: 4,135 citations

SEARCH STRATEGIES

Database: Ovid MEDLINE(R) ALL <1946 to April 05, 2024>

#	Query	Results from 8 Apr 2024
1	exp brain injuries/	84,942
2	Craniocerebral Trauma/	23,904
3	exp Head Injuries, Closed/	15,872
4	exp Skull Fractures/	23,467
5	mTBI*2.tw.	4,114
6	tbi*2.tw.	36,323
7	concuss*.tw.	12,430
8	((head* or cerebr* or crani* or capitis* or brain* or forebrain* or skull* or hemispher* or intracran* or orbit*) adj2 (injur* or trauma* or lesion* or damag* or wound* or destruction* or swell* or oedema* or edema* or fracture* or contusion* or commotion* or pressur*)).tw.	241,005
9	((brain* or cerebr* or intracerebr* or crani* or intracran* or head* or subarachnoid* or subdural* or epidural* or extradural*) adj (haematoma* or hematoma* or hemorrhag* or haemorrhag* or pressur* or bleed*)).tw.	109,661
10	or/1-9	368,353
11	exp Spinal Cord Injuries/	56,925
12	exp Central Cord Syndrome/	145
13	(myelopathy adj3 (traumatic or post-traumatic)).tw,kf.	149
14	((spine or spinal) adj3 (fracture* or wound* or trauma* or injur* or damag*)).tw,kf.	73,696
15	(spinal cord adj3 (contusion* or laceration* or transaction* or trauma* or ischemi*)).tw,kf.	10,362
16	SCI.tw,kf.	44,055
17	exp Paraplegia/	13,619
18	exp Quadriplegia/	8,498
19	(paraplegia* or quadriplegia* or tetraplegia*).tw,kf.	21,750
20	Spinal Cord Compression/	11,977
21	exp Cervical Vertebrae/in	8,470
22	central spinal cord syndrome.tw,kf.	11
23	central cord injury syndrome.tw,kf.	2
24	or/11-23	141,927
25	Brain Death/	9,611
26	(brain adj2 (death or dead)).tw,kf.	11,010
27	((vegetat* or unawareness* or "minimally conscious") adj2 state*1).tw,kf.	4,326
28	(prolonged adj2 unawareness*).tw,kf.	11
29	25 or 26 or 27 or 28	18,877
30	(central nervous system adj2 trauma*).tw,kf.	450

31	(CNS adj2 trauma*).tw,kf.	689
32	10 or 24 or 29 or 30 or 31	516,552
33	exp Cognition/	206,780
34	exp Cognition Disorders/	120,438
35	neurocognit*.tw,kf.	32,095
36	(cognitive or cognition).tw,kf.	547,976
37	Executive Function/	20,290
38	(executive adj2 (function* or control*)).tw,kf.	43,077
39	exp Arousal/	134,199
40	arous*.tw,kf.	46,807
41	attention*.tw,kf.	604,528
42	vigilan*.tw,kf.	27,828
43	or/33-42	1,332,739
44	32 and 43	45,280
45	(dementi* or alzheim*).tw,kf.	292,731
46	exp dementia/	213,631
47	45 or 46	335,329
48	32 and 47	12,213
49	exp clinical trial/	991,128
50	exp Clinical Trials as Topic/	390,465
51	multicenter studies as topic/	24,161
52	(randomi?ed adj7 trial*).tw,kf.	547,543
53	(controlled adj3 trial*).tw,kf.	390,180
54	(clinical adj2 trial*).tw,kf.	529,062
55	((single or doubl* or tripl* or treb*) and (blind* or mask*)).tw,kf.	232,825
56	("4 arm" or "four arm").tw,kf.	1,670
57	or/49-56	1,821,693
58	Case-Control Studies/	332,839
59	Control Groups/	2,121
60	Matched-Pair Analysis/	5,192
61	retrospective studies/	1,192,752
62	((case* adj5 control*) or (case adj3 comparison*) or control group*).tw,kf.	802,897
63	or/58-62	2,112,236
64	cohort studies/	339,626
65	longitudinal studies/	170,572
66	follow-up studies/	696,399
67	prospective studies/	684,096
68	cohort.tw,kf.	849,410
69	longitudinal.tw,kf.	348,596
70	prospective.tw,kf.	753,511
71	retrospective.tw,kf.	805,030

72	or/64-71	3,053,182
73	controlled before-after studies/ or interrupted time series analysis/	2,745
74	57 or 63 or 72 or 73	5,497,589
75	44 and 74	14,198
76	48 and 74	2,875
77	75 or 76	15,693
78	limit 77 to english language	15,091
79	(201612* or 2017* or 2018* or 2019* or 202*).dt,ez,da.	11,249,056
80	78 and 79	7,291

Database: Embase <1974 to 2024 April 05>

#	Query	Results from 8 Apr 2024
1	exp brain injury/	220,790
2	head injury/	53,882
3	mTBI*2.tw.	6,425
4	tbi*2.tw.	59,249
5	concuss*.tw.	16,805
6	((head* or cerebr* or crani* or capitis* or brain* or forebrain* or skull* or hemispher* or intracran* or orbit*) adj2 (injur* or trauma* or lesion* or damag* or wound* or destruction* or swell* or oedema* or edema* or fracture* or contusion* or commotion* or pressur*)).tw.	322,614
7	((brain* or cerebr* or intracerebr* or crani* or intracran* or head* or subarachnoid* or subdural* or epidural* or extradural*) adj (haematoma* or hematoma* or hemorrhag* or haemorrhag* or pressur* or bleed*)).tw.	153,780
8	or/1-7	535,488
9	exp spinal cord injury/	93,514
10	spinal cord ischemia/	5,298
11	(myelopathy adj3 (traumatic or post-traumatic)).tw,kf.	176
12	((spine or spinal) adj3 (fracture* or wound* or trauma* or injur* or damag*)).tw,kf.	94,862
13	(spinal cord adj3 (contusion* or laceration* or transaction* or trauma* or ischemi*)).tw,kf.	13,492
14	SCI.tw,kf.	63,066
15	paraplegia/	26,769
16	quadriplegia/	21,102
17	(paraplegia* or quadriplegia* or tetraplegia*).tw,kf.	27,982
18	central spinal cord syndrome.tw,kf.	12
19	central cord injury syndrome.tw,kf.	4
20	or/9-19	202,545
21	brain death/	16,935
22	(brain adj2 (death or dead)).tw,kf.	17,203

23	((vegetat* or unawareness* or "minimally conscious") adj2 state*1).tw,kf.	6,049
24	(prolonged adj2 unawareness*).tw,kf.	14
25	21 or 22 or 23 or 24	28,524
26	(central nervous system adj2 trauma*).tw,kf.	558
27	(CNS adj2 trauma*).tw,kf.	886
28	8 or 20 or 25 or 26 or 27	743,760
29	exp cognition/	3,092,039
30	cognitive defect/	225,244
31	neurocognit*.tw,kf.	48,033
32	(cognitive or cognition).tw,kf.	748,913
33	executive function/	58,704
34	(executive adj2 (function* or control*)).tw,kf.	61,244
35	arousal/	49,344
36	arous*.tw,kf.	62,231
37	attention*.tw,kf.	743,282
38	vigilan*.tw,kf.	38,658
39	or/29-38	3,983,422
40	exp dementia/	461,234
41	(dementi* or alzheim*).tw,kf.	404,373
42	40 or 41	538,513
43	39 or 42	4,293,606
44	28 and 43	150,111
45	exp clinical trial/	1,894,829
46	exp "clinical trial (topic)"/	460,018
47	(randomi?ed adj7 trial*).tw,kf.	750,641
48	(controlled adj3 trial*).tw,kf.	512,477
49	(clinical adj2 trial*).tw,kf.	766,686
50	((single or doubl* or tripl* or treb*) and (blind* or mask*)).tw,kf.	333,270
51	("4 arm" or "four arm").tw,kf.	2,362
52	exp case control study/	233,931
53	control group/	110,763
54	clinical study/ or retrospective study/	1,756,207
55	((case* adj5 control*) or (case adj3 comparison*) or control group*).tw,kf.	1,138,142
56	cohort analysis/	1,142,835
57	longitudinal study/	210,100
58	follow up/	2,169,162
59	prospective study/	912,538
60	cohort.tw,kf.	1,431,145
61	longitudinal.tw,kf.	471,858
62	prospective.tw,kf.	1,153,561
63	retrospective.tw,kf.	1,334,636

64	or/45-63	8,495,372
65	44 and 64	47,701
66	limit 65 to english language	46,335
67	limit 66 to embase	27,484
68	limit 67 to dc=20161201-20240408	14,205

Database: Cochrane Central Register of Controlled Trials <2014 to Present>

#	Query	Results from 8 Apr 2024
1	exp brain injuries/	3,640
2	Craniocerebral Trauma/	448
3	exp Head Injuries, Closed/	751
4	exp Skull Fractures/	433
5	mTBI*2.tw.	473
6	tbi*2.tw.	3,915
7	concuss*.tw.	1,011
8	((head* or cerebr* or crani* or capitis* or brain* or forebrain* or skull* or hemispher* or intracran* or orbit*) adj2 (injur* or trauma* or lesion* or damag* or wound* or destruction* or swell* or oedema* or edema* or fracture* or contusion* or commotion* or pressur*)).tw.	14,928
9	((brain* or cerebr* or intracerebr* or crani* or intracran* or head* or subarachnoid* or subdural* or epidural* or extradural*) adj (haematoma* or hematoma* or hemorrhag* or haemorrhag* or pressur* or bleed*)).tw.	10,677
10	or/1-9	25,819
11	exp Spinal Cord Injuries/	2,442
12	exp Central Cord Syndrome/	6
13	(myelopathy adj3 (traumatic or post-traumatic)).tw,kw.	1
14	((spine or spinal) adj3 (fracture* or wound* or trauma* or injur* or damag*)).tw,kw.	6,718
15	(spinal cord adj3 (contusion* or laceration* or transaction* or trauma* or ischemi*)).tw,kw.	476
16	SCI.tw,kw.	2,783
17	exp Paraplegia/	277
18	exp Quadriplegia/	243
19	(paraplegia* or quadriplegia* or tetraplegia*).tw,kf.	780
20	Spinal Cord Compression/	148
21	exp Cervical Vertebrae/in	7
22	central spinal cord syndrome.tw,kw.	1
23	central cord injury syndrome.tw,kw.	0
24	or/11-23	8,314
25	Brain Death/	124
26	(brain adj2 (death or dead)).tw,kw.	510

27	((vegetat* or unawareness* or "minimally conscious") adj2 state*1).tw,kw.	284
28	(prolonged adj2 unawareness*).tw,kw.	0
29	25 or 26 or 27 or 28	814
30	(central nervous system adj2 trauma*).tw,kw.	6
31	(CNS adj2 trauma*).tw,kw.	10
32	10 or 24 or 29 or 30 or 31	34,326
33	exp Cognition/	16,081
34	exp Cognition Disorders/	8,282
35	neurocognit*.tw,kw.	5,330
36	(cognitive or cognition).tw,kw.	102,251
37	Executive Function/	1,826
38	(executive adj2 (function* or control*)).tw,kw.	8,918
39	exp Arousal/	10,656
40	arous*.tw,kw.	7,289
41	attention*.tw,kw.	38,836
42	vigilan*.tw,kw.	2,990
43	or/33-42	145,476
44	32 and 43	5,048
45	(dementi* or alzheim*).tw,kw.	24,446
46	exp dementia/	9,216
47	45 or 46	25,456
48	32 and 47	716
49	44 or 48	5,309
50	limit 49 to english language	5,222
51	(2016-12* or 2017* or 2018* or 2019* or 202*).dl.	1,201,099
52	50 and 51	4,046

Database: APA PsycInfo <1806 to March Week 4 2024>

#	Query	Results from 8 Apr 2024
1	exp traumatic brain injury/	23,895
2	exp head injuries/	8,155
3	mTBI*2.tw.	2,626
4	tbi*2.tw.	13,660
5	concuss*.tw.	4,942
6	((head* or cerebr* or crani* or capitis* or brain* or forebrain* or skull* or hemispher* or intracran* or orbit*) adj2 (injur* or trauma* or lesion* or damag* or wound* or destruction* or swell* or oedema* or edema* or fracture* or contusion* or commotion* or pressur*)).tw.	66,877

7	((brain* or cerebr* or intracerebr* or crani* or intracran* or head* or subarachnoid* or subdural* or epidural* or extradural*) adj (haematoma* or hematoma* or hemorrhag* or haemorrhag* or pressur* or bleed*)).tw.	6,590
8	or/1-7	73,337
9	exp spinal cord injuries/	6,890
10	exp Spinal Cord/ and exp Ischemia/	79
11	(myelopathy adj3 (traumatic or post-traumatic)).tw.	8
12	((spine or spinal) adj3 (fracture* or wound* or trauma* or injur* or damag*)).tw.	8,330
13	(spinal cord adj3 (contusion* or laceration* or transaction* or trauma* or ischemi*)).tw.	1,223
14	SCI.tw.	5,572
15	paraplegia/	714
16	quadriplegia/	246
17	(paraplegia* or quadriplegia* or tetraplegia*).tw.	1,738
18	central spinal cord syndrome.tw.	0
19	central cord injury syndrome.tw.	0
20	or/9-19	12,513
21	(brain adj2 (death or dead)).tw.	752
22	((vegetat* or unawareness* or "minimally conscious") adj2 state*1).tw.	1,483
23	(prolonged adj2 unawareness*).tw.	3
24	21 or 22 or 23	2,191
25	(central nervous system adj2 trauma*).tw.	79
26	(CNS adj2 trauma*).tw.	150
27	8 or 20 or 24 or 25 or 26	86,256
28	cognition/	38,251
29	cognitive impairment/	46,024
30	neurocognit*.tw.	20,263
31	(cognitive or cognition).tw.	569,392
32	exp executive function/	25,433
33	(executive adj2 (function* or control*)).tw.	40,531
34	physiological arousal/	9,012
35	exp attention/	83,762
36	arous*.tw.	44,063
37	attention*.tw.	334,669
38	vigilan*.tw.	12,415
39	or/28-38	916,080
40	(dementi* or alzheim*).tw.	124,140
41	exp dementia/	95,177
42	alzheimer's disease/	57,624
43	40 or 41 or 42	125,479
44	39 or 43	983,391

45	27 and 44	26,775
46	clinical trials/	12,325
47	(randomi?ed adj7 trial*).tw.	76,202
48	(controlled adj3 trial*).tw.	61,548
49	(clinical adj2 trial*).tw.	45,238
50	((single or doubl* or tripl* or treb*) and (blind* or mask*)).tw.	33,253
51	("4 arm" or "four arm").tw.	197
52	experiment controls/	966
53	retrospective studies/	988
54	((case* adj5 control*) or (case adj3 comparison*) or control group*).tw.	119,702
55	exp Longitudinal Studies/	17,471
56	longitudinal studies/	16,143
57	Followup Studies/	12,402
58	cohort.tw.	91,151
59	longitudinal.tw.	151,232
60	prospective.tw.	74,045
61	retrospective.tw.	45,775
62	or/46-61	531,901
63	45 and 62	4,801
64	limit 63 to english language	4,634
65	limit 64 to up=20161201-20240408	2,027

Database: SCOPUS
Date Searches: April 8, 2024

((((TITLE-ABS-KEY ("central spinal cord syndrome")) OR (TITLE-ABS-KEY ("central cord injury syndrome")) OR ((TITLE-ABS-KEY (mtbi* OR tbi* OR concuss*)) OR (TITLE-ABS-KEY (((head* OR cerebr* OR crani* OR capitis* OR brain* OR forebrain* OR skull* OR hemispher* OR intracran* OR orbit*) near/2 (injur* OR trauma* OR lesion* OR damag* OR wound* OR destruction* OR swell*)))) OR (TITLE-ABS-KEY (((head* OR cerebr* OR crani* OR capitis* OR brain* OR forebrain* OR skull* OR hemispher* OR intracran* OR orbit*) near/2 (oedema* OR edema* OR fracture* OR contusion* OR commotion* OR pressur*)))) OR (TITLE-ABS-KEY (((brain* OR cerebr* OR intracerebr* OR crani* OR intracran* OR head* OR subarachnoid* OR subdural* OR epidural* OR extradural*) near/2 (haematoma* OR hematoma* OR hemorrhag* OR haemorrhag* OR pressur* OR bleed*)))) OR (TITLE-ABS-KEY ((myelopathy AND near/3 (traumatic OR post-traumatic)))) OR (TITLE-ABS-KEY (((spine OR spinal) near/3 (fracture* OR wound* OR trauma* OR injur* OR damag*)))) OR (TITLE-ABS-KEY ((spinal AND cord AND near/3 (contusion* OR laceration* OR transaction* OR trauma* OR ischemi*)))) OR (TITLE-ABS-KEY ((paraplegia* OR quadriplegia* OR tetraplegia*))))) OR (TITLE-ABS-KEY ((brain AND near/2 (death OR dead)))) OR (TITLE-ABS-KEY (((vegetat* OR unawareness* OR "minimally conscious") near/2 AND state*))) OR (TITLE-ABS-KEY ((prolonged AND near/2 AND unawareness*))) OR (TITLE-ABS-KEY ((central AND nervous AND system AND near/2 AND trauma*))) OR (TITLE-

ABS-KEY ((cns AND near/2 AND trauma*)))) AND (((TITLE-ABS-KEY (cognitive OR cognition OR neurocogniti*) OR TITLE-ABS-KEY ((executive AND near/2 (function* OR control*))))) OR (TITLE-ABS-KEY (arous* OR attention* OR vigilan* OR dementi* OR alzheim*)))) AND (((TITLE-ABS-KEY ((controlled AND near/3 AND trial*)) OR TITLE-ABS-KEY ((clinical AND near/2 AND trial*)) OR TITLE-ABS-KEY (((single OR doubl* OR tripl* OR treb*) AND (blind* OR mask*))) OR TITLE-ABS-KEY (("4 arm" OR "four arm")) OR TITLE-ABS-KEY ((case* AND near/5 AND control*)) OR TITLE-ABS-KEY ((case AND near/3 AND comparison*)) OR TITLE-ABS-KEY (control AND group*) OR TITLE-ABS-KEY (cohort* OR longitudinal OR prospective OR retrospective))) OR (TITLE-ABS-KEY (random*)) OR (TITLE-ABS-KEY ((randomized AND near/7 AND trial*) OR (randomised AND near/7 AND trial*))))) AND ORIG-LOAD-DATE > 20161201 AND (LIMIT-TO (LANGUAGE , "English"))

Results: 4,135 citations
