# Supplementary material for: A PROGRESS-driven approach to cognitive outcomes after traumatic brain injury: A study protocol for advancing equity, diversity, and inclusion through knowledge synthesis and mobilization
Source: PLoS One. 2024 Jul 22;19(7):e0307418. doi: 10.1371/journal.pone.0307418 (PMC11262676; doi:10.1371/journal.pone.0307418)
Supplement: S3 File — (PDF) [file pone.0307418.s004.pdf]

Supplementary File 4: Search strategies for update of systematic review on sex and gender effects in clinical and functional outcomes of traumatic brain injury

SEARCH DETAILS

Completed by: Cynthia Chui, MI
Submitted: March 26, 2024

Searches were conducted in Cochrane Central Register of Controlled Trials (Ovid), CINAHL Ultimate (EBSCOhost), Embase (Ovid), Ovid MEDLINE (Epub Ahead of Print, In-Process & Other Non-Indexed Citations, Ovid MEDLINE(R) Daily and Ovid MEDLINE(R)), PsycINFO (Ovid), and Web of Science for an update of a systematic review (doi: 10.3389/fneur.2021.678971).

Search strategies were created by Jessica Babineau for the previous systematic review, which included the use of text words and subject headings (e.g. MeSH, Emtree) related to (1) traumatic brain injury and (2) sex or (3) gender. Searches were limited to human studies, and English language papers when possible. Case reports were excluded when possible. All searches were updated to include articles added to the database from September 2019 to March 26, 2024.

The updated search strategies included minor revisions (e.g. due to updates to databases):

- The search field for “kw” was changed to “kf” in Embase
- The search field “id” was added for PsycInfo, and “AK” for Web of Science to search for author keywords, where appropriate and consistent with the original Medline strategy from which it was translated from
- CINAHL changed to CINAHL Ultimate (broader coverage); specifying “TI” or “AB” in the search strategy, where appropriate and consistent with the original Medline strategy
- Truncation added for concuss* and postconcuss* in the Web of Science strategy

UPDATED SEARCH – NEW RESULTS PRE-DUPLICATE REMOVAL

TOTAL Results: 6839 citations

- Cochrane Central Register of Controlled Trials: 1292 citations
- CINAHL Ultimate: 573 citations
- Embase: 1754 citations
- MEDLINE: 1316 citations
- PsycINFO: 351 citations
- Web of Science: 1553 citations

<u>SEARCH STRATEGIES</u>

Database: Ovid MEDLINE: Epub Ahead of Print, In-Process & Other Non-Indexed Citations, Ovid MEDLINE(R) Daily and Ovid MEDLINE(R) <1946-Present>

#	Query	Results from 25 Mar 2024
1	Brain Injuries/	57,218
2	exp Brain Injuries, Traumatic/	25,933
3	exp Brain Concussion/	12,824
4	Craniocerebral Trauma/	23,892
5	tbi*2.tw,kf.	36,995
6	mtbi*2.tw,kf.	4,338
7	wrTBI*2.tw,kf.	17
8	concuss*.tw,kf.	13,642
9	postconcuss*.tw,kf.	1,754
10	((head* or cerebr* or crani* or skull* or intracran*) adj2 (injur* or trauma* or damag* or wound* or swell* or oedema* or edema* or fracture* or contusion* or pressur*)).tw,kf.	102,578
11	((brain* or cerebr* or intracerebr* or crani* or intracran* or head* or subdural* or epidural* or extradural*) adj (haematoma* or hematoma* or hemorrhag* or haemorrhag* or bleed*)).tw,kf.	66,824
12	or/1-11	241,567
13	Sex Characteristics/	62,244
14	exp sex distribution/	66,601
15	exp Gender Identity/	27,958
16	(sex adj2 (differenc* or charact*)).tw,kf.	67,766
17	sex-related.tw,kf.	8,899
18	sex-based.tw,kf.	3,211
19	(sexes adj2 (differenc* or charact*)).tw,kf.	2,759
20	(gender adj2 (differenc* or charact*)).tw,kf.	53,107
21	gender-related.tw,kf.	6,249
22	gender-based.tw,kf.	5,861
23	(male? or men or man or masculin*).ti,kf.	343,391
24	(female? or women or woman or feminin*).ti,kf.	489,153
25	sex.ti,kf.	148,257
26	sexes.ti,kf.	1,862
27	gender*.ti,kf.	81,068
28	or/13-27	1,104,451
29	12 and 28	4,961
30	29 not (exp animals/ not exp humans/)	4,548
31	case reports/	2,391,969
32	30 not 31	3,968

33	limit 32 to english language	3,706
34	(201909* or 20191* or 202*).dt,ez,da.	7,705,588
35	33 and 34	1,316

Database: Cochrane Central Register of Controlled Trials <2014 to Present>

#	Query	Results from 25 Mar 2024
1	Brain Injuries/	2,501
2	exp Brain Injuries, Traumatic/	1,638
3	exp Brain Concussion/	660
4	Craniocerebral Trauma/	446
5	tbi*2.tw,kw.	3,891
6	mtbi*2.tw,kw.	470
7	wrTBI*2.tw,kw.	0
8	concuss*.tw,kw.	1,068
9	postconcuss*.tw,kw.	319
10	((head* or cerebr* or crani* or skull* or intracran*) adj2 (injur* or trauma* or damag* or wound* or swell* or oedema* or edema* or fracture* or contusion* or pressur*)).tw,kw.	6,969
11	((brain* or cerebr* or intracerebr* or crani* or intracran* or head* or subdural* or epidural* or extradural*) adj (haematoma* or hematoma* or hemorrhag* or haemorrhag* or bleed*)).tw,kw.	9,532
12	or/1-11	21,212
13	Sex Characteristics/	1,650
14	exp sex distribution/	982
15	exp Gender Identity/	380
16	(sex adj2 (differenc* or charact*)).tw,kw.	6,515
17	sex-related.tw,kw.	359
18	sex-based.tw,kw.	160
19	(sexes adj2 (differenc* or charact*)).tw,kw.	89
20	(gender adj2 (differenc* or charact*)).tw,kw.	3,028
21	gender-related.tw,kw.	242
22	gender-based.tw,kw.	313
23	(male? or men or man or masculin*).ti,kw.	426,415
24	(female? or women or woman or feminin*).ti,kw.	496,189
25	sex.ti,kw.	11,056
26	sexes.ti,kw.	33
27	gender*.ti,kw.	15,281
28	or/13-27	569,609
29	12 and 28	7,146
30	29 not (exp animals/ not exp humans/)	7,132

31	case reports/	0
32	30 not 31	7,132
33	limit 32 to english language	6,982
34	limit 33 to conference proceeding	2,585
35	33 not 34	4,397
36	(2019-09* or 2019-1* or 2022* or 2023* or 2024*).dl.	341,603
37	35 and 36	1,292

Database: Embase <1974 to 2024 March 22>

#	Query	Results from 25 Mar 2024
1	exp traumatic brain injury/	70,114
2	brain injury/	98,960
3	exp concussion/	16,737
4	head injury/	53,815
5	tbi*2.tw,kf.	60,091
6	mtbi*2.tw,kf.	6,955
7	wrTBI*2.tw,kf.	24
8	concuss*.tw,kf.	18,566
9	postconcuss*.tw,kf.	2,335
10	((head* or cerebr* or crani* or skull* or intracran*) adj2 (injur* or trauma* or damag* or wound* or swell* or oedema* or edema* or fracture* or contusion* or pressur*)).tw,kf.	132,341
11	((brain* or cerebr* or intracerebr* or crani* or intracran* or head* or subdural* or epidural* or extradural*) adj (haematoma* or hematoma* or hemorrhag* or haemorrhag* or bleed*)).tw,kf.	96,812
12	or/1-11	390,358
13	"gender and sex"/	1,262
14	gender identity/	23,065
15	(sex adj2 (differenc* or charact*)).tw,kf.	87,082
16	sex-related.tw,kf.	10,935
17	sex-based.tw,kf.	4,251
18	(sexes adj2 (differenc* or charact*)).tw,kf.	3,619
19	(gender adj2 (differenc* or charact*)).tw,kf.	76,946
20	gender-related.tw,kf.	8,328
21	gender-based.tw,kf.	7,167
22	(male? or men or man or masculin*).ti,kf.	377,045
23	(female? or women or woman or feminin*).ti,kf.	592,436
24	sex.ti,kf.	165,307
25	sexes.ti,kf.	1,838
26	gender*.ti,kf.	108,989

27	or/13-26	1,209,704
28	12 and 27	7,074
29	28 not ((exp animals/ or exp animal experimentation/ or nonhuman/) not exp human/)	5,859
30	limit 29 to english language	5,506
31	case study/	99,911
32	30 not 31	5,476
33	limit 32 to medline	684
34	32 not 33	4,792
35	limit 34 to dc=20190901-20240325	1,754

Database: APA PsycInfo <1806 to March Week 4 2024>

#	Query	Results from 25 Mar 2024
1	exp traumatic brain injury/	23,895
2	head injuries/	4,842
3	tbi*2.tw	13,660
4	mtbi*2.tw	2,626
5	wrTBI*2.tw	9
6	concuss*.tw	4,942
7	postconcuss*.tw	1,027
8	((head* or cerebr* or crani* or skull* or intracran*) adj2 (injur* or trauma* or damag* or wound* or swell* or oedema* or edema* or fracture* or contusion* or pressur*)).tw	13,850
9	((brain* or cerebr* or intracerebr* or crani* or intracran* or head* or subdural* or epidural* or extradural*) adj (haematoma* or hematoma* or hemorrhag* or haemorrhag* or bleed*)).tw	4,050
10	or/1-9	39,416
11	human sex differences/	127,571
12	exp gender identity/	49,814
13	(sex adj2 (differenc* or charact*)).tw	42,965
14	sex-related.tw	2,553
15	sex-based.tw	779
16	(sexes adj2 (differenc* or charact*)).tw	561
17	(gender adj2 (differenc* or charact*)).tw	56,977
18	gender-related.tw	3,676
19	gender-based.tw	4,464
20	(male? or men or man or masculin*).ti,id.	218,106
21	(female? or women or woman or feminin*).ti,id.	239,935
22	sex.ti,id.	103,652
23	sexes.ti,id.	705

24	gender*.ti,id.	107,232
25	or/11-24	600,099
26	10 and 25	2,071
27	limit 26 to ("0200 book" or "0240 authored book" or "0280 edited book" or "0300 encyclopedia")	69
28	exp case report/	23,405
29	26 not (27 or 28)	1,673
30	limit 29 to english language	1,600
31	limit 30 to up=20190901-20240325	351

Database: CINAHL Ultimate
Searched: March 25, 2024

#	Query	Limiters/Expanders	Last Run Via	Results
S1	(MH "Brain Injuries+")	Expanders - Apply equivalent subjects Search modes - Boolean/Phrase	Interface - EBSCOhost Research Databases Search Screen - Advanced Search Database - CINAHL Ultimate	34,219
S2	(MH "Brain Concussion+")	Expanders - Apply equivalent subjects Search modes - Boolean/Phrase	Interface - EBSCOhost Research Databases Search Screen - Advanced Search Database - CINAHL Ultimate	6,824
S3	(MH "Head Injuries")	Expanders - Apply equivalent subjects Search modes - Boolean/Phrase	Interface - EBSCOhost Research Databases Search Screen - Advanced Search Database - CINAHL Ultimate	8,329
S4	TI (TBI##) OR AB (TBI##)	Expanders - Apply equivalent subjects Search modes - Boolean/Phrase	Interface - EBSCOhost Research Databases Search Screen - Advanced Search Database - CINAHL Ultimate	10,692
S5	TI (mTBI##) OR AB (mTBI##)	Expanders - Apply equivalent subjects Search modes - Boolean/Phrase	Interface - EBSCOhost Research Databases Search Screen - Advanced Search Database - CINAHL Ultimate	1,445
S6	TI (wrTBI##) OR AB (wrTBI##)	Expanders - Apply equivalent subjects	Interface - EBSCOhost Research Databases Search Screen - Advanced	10

		Search modes - Boolean/Phrase	Search<br>Database - CINAHL Ultimate	
S7	TI (concuss*) OR AB (concuss*)	Expanders - Apply equivalent subjects<br>Search modes - Boolean/Phrase	Interface - EBSCOhost Research Databases<br>Search Screen - Advanced Search<br>Database - CINAHL Ultimate	6,803
S8	TI (postconcuss*) OR AB (postconcuss*)	Expanders - Apply equivalent subjects<br>Search modes - Boolean/Phrase	Interface - EBSCOhost Research Databases<br>Search Screen - Advanced Search<br>Database - CINAHL Ultimate	925
S9	TI ((head* or cerebr* or crani* or skull* or intracran*) n2 (injur* or trauma* or damag* or wound* or swell* or oedema* or edema* or fracture* or contusion* or pressur*)) OR AB ((head* or cerebr* or crani* or skull* or intracran*) n2 (injur* or trauma* or damag* or wound* or swell* or oedema* or edema* or fracture* or contusion* or pressur*))	Expanders - Apply equivalent subjects<br>Search modes - Boolean/Phrase	Interface - EBSCOhost Research Databases<br>Search Screen - Advanced Search<br>Database - CINAHL Ultimate	24,667
S10	TI ((brain* or cerebr* or intracerebr* or crani* or intracran* or head* or subdural* or epidural* or extradural*) n1 (haematoma* or hematoma* or hemorrhag* or haemorrhag* or bleed*)) OR AB ((brain* or cerebr* or intracerebr* or crani* or intracran* or head* or subdural* or epidural* or extradural*) n1 (haematoma* or hematoma* or hemorrhag* or haemorrhag* or bleed*))	Expanders - Apply equivalent subjects<br>Search modes - Boolean/Phrase	Interface - EBSCOhost Research Databases<br>Search Screen - Advanced Search<br>Database - CINAHL Ultimate	15,523
S11	S1 OR S2 OR S3 OR S4 OR S5 OR S6 OR S7 OR S8 OR S9 OR S10	Expanders - Apply equivalent subjects	Interface - EBSCOhost Research Databases<br>Search Screen - Advanced	73,362

		Search modes - Boolean/Phrase	Search<br>Database - CINAHL Ultimate	
S12	(MH "Gender Identity+")	Expanders - Apply equivalent subjects<br>Search modes - Boolean/Phrase	Interface - EBSCOhost Research Databases<br>Search Screen - Advanced Search<br>Database - CINAHL Ultimate	10,157
S13	TI (sex n2 (differenc* or charact*)) OR AB (sex n2 (differenc* or charact*))	Expanders - Apply equivalent subjects<br>Search modes - Boolean/Phrase	Interface - EBSCOhost Research Databases<br>Search Screen - Advanced Search<br>Database - CINAHL Ultimate	17,294
S14	TI (sex-related) OR AB (sex-related)	Expanders - Apply equivalent subjects<br>Search modes - Boolean/Phrase	Interface - EBSCOhost Research Databases<br>Search Screen - Advanced Search<br>Database - CINAHL Ultimate	1,825
S15	TI (sex-based) OR AB (sex-based)	Expanders - Apply equivalent subjects<br>Search modes - Boolean/Phrase	Interface - EBSCOhost Research Databases<br>Search Screen - Advanced Search<br>Database - CINAHL Ultimate	993
S16	TI (sexes n2 (differenc* or charact*)) OR AB (sexes n2 (differenc* or charact*))	Expanders - Apply equivalent subjects<br>Search modes - Boolean/Phrase	Interface - EBSCOhost Research Databases<br>Search Screen - Advanced Search<br>Database - CINAHL Ultimate	17,294
S17	TI (gender n2 (differenc* or charact*)) OR AB (gender n2 (differenc* or charact*))	Expanders - Apply equivalent subjects<br>Search modes - Boolean/Phrase	Interface - EBSCOhost Research Databases<br>Search Screen - Advanced Search<br>Database - CINAHL Ultimate	23,176
S18	TI (gender-related) OR AB (gender-related)	Expanders - Apply equivalent subjects<br>Search modes - Boolean/Phrase	Interface - EBSCOhost Research Databases<br>Search Screen - Advanced Search<br>Database - CINAHL Ultimate	1,803
S19	TI (male# or men or man or masculin*)	Expanders - Apply equivalent subjects<br>Search modes - Boolean/Phrase	Interface - EBSCOhost Research Databases<br>Search Screen - Advanced Search<br>Database - CINAHL Ultimate	88,918

S20	TI (female# or women or woman or feminin*)	Expanders - Apply equivalent subjects<br>Search modes - Boolean/Phrase	Interface - EBSCOhost Research Databases<br>Search Screen - Advanced Search<br>Database - CINAHL Ultimate	222,940
S21	TI (sex or sexes or gender*)	Expanders - Apply equivalent subjects<br>Search modes - Boolean/Phrase	Interface - EBSCOhost Research Databases<br>Search Screen - Advanced Search<br>Database - CINAHL Ultimate	69,117
S22	S12 OR S13 OR S14 OR S15 OR S16 OR S17 OR S18 OR S19 OR S20 OR S21	Expanders - Apply equivalent subjects<br>Search modes - Boolean/Phrase	Interface - EBSCOhost Research Databases<br>Search Screen - Advanced Search<br>Database - CINAHL Ultimate	374,138
S23	S11 AND S22	Expanders - Apply equivalent subjects<br>Search modes - Boolean/Phrase	Interface - EBSCOhost Research Databases<br>Search Screen - Advanced Search<br>Database - CINAHL Ultimate	1,626
S24	PT (case study)	Expanders - Apply equivalent subjects<br>Search modes - Boolean/Phrase	Interface - EBSCOhost Research Databases<br>Search Screen - Advanced Search<br>Database - CINAHL Ultimate	449,333
S25	TI (case n1 (study or report))	Expanders - Apply equivalent subjects<br>Search modes - Boolean/Phrase	Interface - EBSCOhost Research Databases<br>Search Screen - Advanced Search<br>Database - CINAHL Ultimate	118,802
S26	(MH "Case Studies")	Expanders - Apply equivalent subjects<br>Search modes - Boolean/Phrase	Interface - EBSCOhost Research Databases<br>Search Screen - Advanced Search<br>Database - CINAHL Ultimate	27,900
S27	S24 OR S25 OR S26	Expanders - Apply equivalent subjects<br>Search modes - Boolean/Phrase	Interface - EBSCOhost Research Databases<br>Search Screen - Advanced Search<br>Database - CINAHL Ultimate	517,068
S28	S23 NOT S27	Expanders - Apply equivalent subjects<br>Search modes - Boolean/Phrase	Interface - EBSCOhost Research Databases<br>Search Screen - Advanced	1,432

			Search<br>Database - CINAHL Ultimate	
S29	S28	Limiters - English Language<br>Expanders - Apply equivalent subjects<br>Search modes - Boolean/Phrase	Interface - EBSCOhost Research Databases<br>Search Screen - Advanced Search<br>Database - CINAHL Ultimate	1,427
S30	(EM 20190901- OR (ZD "in process" AND RD 20190901-))	Limiters - English Language<br>Expanders - Apply equivalent subjects<br>Search modes - Boolean/Phrase	Interface - EBSCOhost Research Databases<br>Search Screen - Advanced Search<br>Database - CINAHL Ultimate	2,306,501
S31	S29 AND S30	Expanders - Apply equivalent subjects<br>Search modes - Boolean/Phrase	Interface - EBSCOhost Research Databases<br>Search Screen - Advanced Search<br>Database - CINAHL Ultimate	573

Database: Web of Science Core Collection
Date Searched: March 25, 2024
(Indexes: WOS.SCI: 1900 to 2024; WOS.AHCI: 1975 to 2024; WOS.BHCI: 2005 to 2024; WOS.BSCI: 2005 to 2024; WOS.ESCI: 2005 to 2024; WOS.ISTP: 1990 to 2024; WOS.SSCI: 1900 to 2024; WOS.ISSHP: 1990 to 2024)

#	Search Query	Results
1	TS=(tbi* OR mTBI* OR wrTBI* OR concuss OR postconcuss)	48329
2	TS=((head* or cerebr* or crani* or skull* or intracran*) NEAR/2 (injur* or trauma* or damag* or wound* or swell* or oedema* or edema* or fracture* or contusion* or pressur*))	132810
3	TS=((brain* or cerebr* or intracerebr* or crani* or intracran* or head* or subdural* or epidural* or extradural*) NEAR/1 (haematoma* or hematoma* or hemorrhag* or haemorrhag* or bleed*))	75346
4	#1 OR #2 OR #3	231009
5	TS=(sex NEAR/2 (differenc* OR charact*))	134669
6	TS=(sex-related)	10803
7	TS=(sex-based)	4421
8	TS=(sexes NEAR/2 (differenc* OR charact*))	134669
9	TS=(gender NEAR/2 (differenc* OR charact*))	149115
10	TS=(gender-related)	9796

11	TS=(gender-based)	12696
12	(TI=(male* OR men OR man OR masculin*)) OR AK=(male* OR men OR man OR masculin*)	638957
13	(TI=(female* OR women OR woman OR feminin*)) OR AK=(female* OR women OR woman OR feminin*)	898347
14	(TI=(sex or sexes)) OR AK=(sex or sexes)	254247
15	#5 OR #6 OR #7 OR #8 OR #9 OR #10 OR #11 OR #12 OR #13 OR #14	1830682
16	#4 AND #15	4222
17	(#16) AND LA=(English)	4114
18	#17 Timespan: 2019-09-01 to 2024-03-25	1553
