# Supplementary material for: A PROGRESS-driven approach to cognitive outcomes after traumatic brain injury: A study protocol for advancing equity, diversity, and inclusion through knowledge synthesis and mobilization
Source: PLoS One. 2024 Jul 22;19(7):e0307418. doi: 10.1371/journal.pone.0307418 (PMC11262676; doi:10.1371/journal.pone.0307418)
Supplement: S4 File — (PDF) [file pone.0307418.s005.pdf]

Informed Consent Form for Participation in a Research Study

Study Title: A PROGRESS-driven approach to cognitive outcomes after traumatic brain injury: a study protocol for advancing equity, diversity, and inclusion through knowledge synthesis and mobilization

Short title: A PROGRESS-driven approach to cognitive outcomes after traumatic brain injury

Study Doctor: Tatyana Mollayeva, MD PhD
KITE-Toronto Rehabilitation Institute, University Health Network
416 597 3422 ext 7848
tatyana.mollayeva@uhn.ca

Please note that communication via e-mail is not absolutely secure. Thus, please do not communicate personal sensitive information via e-mail.

Funder: Canadian Institutes for Health Research Op. Grant: Brain Health and Reduction of Risk for Age-related Cognitive Impairment - KS and Mobilization Grants - Sex and Gender Differences (SGD) (#503552)

Introduction

You are being invited to participate in a research study. You are invited to participate in this study because you have personal and/or professional experience with traumatic brain injury (TBI). This consent form provides you with information to help you make an informed choice. Please read this document carefully and ask any questions you may have. All your questions should be answered to your satisfaction before you decide whether to participate in this research study. You may find it helpful to discuss it with your friends and family.

Please take your time in making your decision.

Taking part in this study is voluntary. You have the option to not participate at all or you may choose to leave the study at any time. Whatever you choose, it will not affect the usual medical care that you receive outside the study.

Is there a conflict of interest?

The KITE-Toronto Rehabilitation Institute is receiving financial payment from the Canadian Institutes for Health Research to cover the cost of conducting this study.

What is the background information for this study?

Traumatic brain injury (TBI) is a common injury that affects people of all walks of life. However, it affects each person differently based on their unique biological and social characteristics. Our research applies a framework called PROGRESS-Plus to explore how factors like **P**lace of residence, **R**ace, **O**ccupation, **G**ender, and others affect brain health after injury.

The image below illustrates the PROGRESS-Plus framework we are using to guide our research.

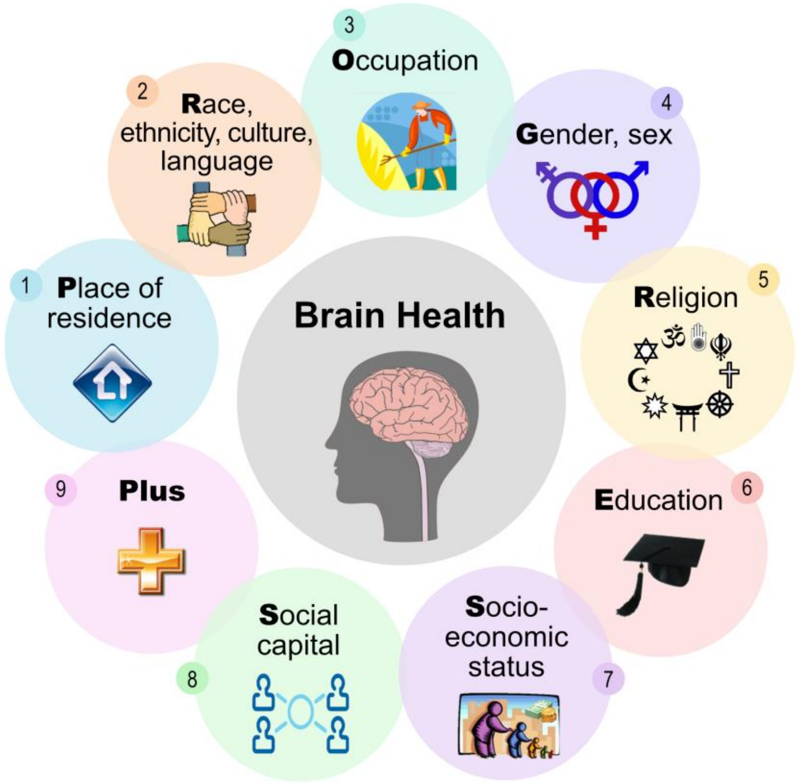

Why is this study being done?
The purpose of this study is to explore how PROGRESS-Plus factors are integrated in TBI research on cognition and brain health. Our study has three main objectives:

1. Organize data on PROGRESS-Plus factors from published research involving people with traumatic brain injury;
2. Understand which factors are most important to people with TBI and people in their support system;
3. Make our research findings accessible to people who may benefit from that knowledge. These people include persons with TBI and people who support them, such as family members, friends, and professionals in the field of TBI.

What other choices are there?
You do not have to take part in this study.

How many people will take part in this study?
It is anticipated that at least 50 people will take part in this study from locations all over the world.

This study should take 2 years to complete and the results should be known in about 1 year.

What are the study procedures?
You will be provided with a survey. The purpose of the survey is to understand which PROGRESS-Plus factors are most important to people with TBI and people who support them, and how to advance knowledge on this topic. The survey will take about 25 to 30 minutes to complete.

The information you provide is for research purposes only. Some of the questions are personal. You can choose not to answer questions if you wish.

How long will participants be in the study?
Your participation involves completion of an online survey, which takes approximately 25 to 30 minutes to complete.

Can participants choose to leave the study?
You can choose to end your participation in this research (called withdrawal) at any time without having to provide a reason. To withdraw, exit the survey before clicking the "Done" button at the end. This way, your data will not be recorded.

If you decide to leave the study after clicking "Done" at the end of the survey, your data will still be included in the analysis. This is because we do not collect any personal information that would allow your survey responses to be identified and have your data removed.

What are the risks or harms of participating in this study?
It is possible that you may get upset by reflecting on challenging personal or professional experiences related to TBI. If you feel any discomfort during the survey, you can skip questions, take a break, or stop answering the survey.

What are the benefits of participating in this study?
You may benefit from the opportunity to share your perspectives on including PROGRESS-Plus factors in TBI research. We anticipate that your participation will help advance knowledge and consideration of PROGRESS-Plus factors in TBI research and practice.

How will participant information be kept confidential?
If you decide to participate in this study, the study staff will only collect the information they need for this study.

Your data will be shared as described in this consent form and/or as required by law and/or applicable research regulations. Records identifying you at this centre will be kept confidential and, to the extent permitted by applicable laws, will not be disclosed or made publicly available.

Authorized representatives of the following organizations may come to the hospital or be given remote access to an electronic portal (via Internet) to look at your study records at the site where these records are held, to check that the information collected for the study is correct and follows proper laws and guidelines. When using the electronic portal, we will share your medical record number using a secure method, so that your records are included as part of their review.

- Representatives of the University Health Network including the UHN Research Ethics Board, who oversees the ethical conduct of this study at UHN

These individuals have completed privacy training and signed confidentiality agreements and/or are required by law to keep your information confidential.

Whether on-site or remotely, UHN makes all efforts to ensure that your information is shared in a way that is secure and private (encrypted). However, any electronic communication carries some risk of third parties gaining unauthorized access to information.

Studies involving humans sometimes collect information on race and ethnicity as well as other

characteristics of individuals because these characteristics may influence people's health outcomes and their experiences in society. Providing information on your race or ethnic origin is voluntary.

If the results of this study are published, your identity will remain confidential. It is expected that the information collected during this study will be used in analyses, published in scientific journals and presented to the scientific community at meetings and conferences.

Even though the likelihood that someone may identify you from the study data is very small, it can never be completely eliminated.

The study doctor will keep any personal health information about you in a secure and confidential location for 5 years.

Data collected uses Survey Monkey and no assurance can be made about its confidentiality or that it will only be used for research purposes. Survey Monkey, collects the IP address of the device a respondent uses to complete the survey. An IP address is a number that identifies a device on the internet. Survey Monkey stores IP addresses for 13 months, however this information is not accessible to the research team. Therefore, the research team will not be able to identify you.

What is the cost to participants?
There are no costs associated with participating in this study.

Are study participants paid to be in this study?
You will not be paid for taking part in this study.

What are the rights of participants in a research study?
You will be told, in a timely manner, about new information that may be relevant to your willingness to stay in this study.

Your rights to privacy are legally protected by federal and provincial laws that require safeguards to ensure that your privacy is respected.

By signing this form you do not give up any of your legal rights against the study doctor, sponsor or involved institutions for compensation, nor does this form relieve the study doctor, sponsor or their agents of their legal and professional responsibilities.

Whom do participants contact for questions?
If you have questions about taking part in this study, or if you suffer a research-related injury, you can talk to the Principal Investigator:

Tatyana Mollayeva	416 597 3422 ext 7848
______________________________	___________________________
Name	Telephone

If you have questions about your rights as a participant or about ethical issues related to this study, call the Chair of the University Health Network Research Ethics Board (UHN REB) or the Research Ethics office number at 416-581-7849. The REB is a group of people who oversee the ethical conduct of research studies. The UHN REB is not involved in the study at all. Everything that you discuss will be kept confidential.

TITLE: **A PROGRESS-driven approach to cognitive outcomes after traumatic brain injury: a study protocol for advancing equity, diversity, and inclusion through knowledge synthesis and mobilization**

CONSENT

- All of my questions have been answered
- I do not give up any legal rights by giving my consent to participate.
- I agree to participate in this study.
