# Supplementary material for: A PROGRESS-driven approach to cognitive outcomes after traumatic brain injury: A study protocol for advancing equity, diversity, and inclusion through knowledge synthesis and mobilization
Source: PLoS One. 2024 Jul 22;19(7):e0307418. doi: 10.1371/journal.pone.0307418 (PMC11262676; doi:10.1371/journal.pone.0307418)
Supplement: S1 Table — (PDF) [file pone.0307418.s006.pdf]

Supplementary Table 2: Attributes of brain health

Brain health attribute	Source
physical health/well-being/functioning	AHA/ASA, Brain health learn and act group
mental health/well-being/functioning	AHA/ASA, Brain health learn and act group
social well-being/functioning	AHA/ASA, Brain health learn and act group
absence of cognitive impairment, neurodegenerative disease, and comorbid disorders that interfere with cognitive functioning	AHA/ASA
state of brain functioning across cognitive, sensory, socio-emotional, behavioral, and motor domains	WHO
utilizing the ability to draw on the strengths of the brain to, for example, remember, learn, concentrate, interact with others and maintain a clear and active mind	Alzheimer’s Association
life-long dynamic state of cognitive, emotional and motor domains underpinned by physiological processes	Chen et al. 2021
**Attributes removed due to constructs being too broad and/or difficult measure**	
preservation of neuronal function to meet demands of life and adapt to one’s environment	AHA/ASA
continuum of life’s stages / lifelong approach to brain health	WHO, Alzheimer’s Association
making the most of the brain’s capacity	Alzheimer’s Association
reducing risk as one ages	Alzheimer’s Association
fully balanced, continuous development and use of the brain	Brain health learn and act group
maintenance of optimal brain structure and function	AHA/ASA

Abbreviations: American Heart Association/ American Stroke Association (AHA/ASA), World Health Organization (WHO)

References:

Gorelick PB, Sorond FA. What is brain health? Cereb Circ Cogn Behav. 2023 Oct 25;6:100190. doi: 10.1016/j.cccb.2023.100190. PMID: 38292017; PMCID: PMC10826122.

Chen Y, Demnitz N, Yamamoto S, Yaffe K, Lawlor B, Leroi I. Defining brain health: A concept analysis. Int J Geriatr Psychiatry. 2021 Apr 30;37(1). doi: 10.1002/gps.5564. Epub ahead of print. PMID: 34131954.
